# Thiamine diphosphate reduction strongly correlates with brain glucose hypometabolism in Alzheimer’s disease, whereas amyloid deposition does not

**DOI:** 10.1186/s13195-018-0354-2

**Published:** 2018-03-01

**Authors:** Shaoming Sang, Xiaoli Pan, Zhichun Chen, Fan Zeng, Shumei Pan, Huimin Liu, Lirong Jin, Guoqiang Fei, Changpeng Wang, Shuhua Ren, Fangyang Jiao, Weiqi Bao, Weiyan Zhou, Yihui Guan, Yiqiu Zhang, Hongcheng Shi, Yanjiang Wang, Xiang Yu, Yun Wang, Chunjiu Zhong

**Affiliations:** 1 Department of Neurology, Zhongshan Hospital, Fudan University, 180 Fenglin Road, Shanghai, 200032 China; 2 Institutes of Brain Science & Collaborative Innovation Center for Brain Science, State Key Laboratory of Medical Neurobiology, Fudan University, Room 1105, Mingdao Building, 138 Yixueyuan Road, Shanghai, 200032 China; 3 Department of Neurology, Daping Hospital, Third Military Medical University, Chongqing, 400042 China; 4 PET Center, Huashan Hospital, Fudan University, Shanghai, 200040 China; 5 Department of Nuclear Medicine, Zhongshan Hospital, Fudan University, Shanghai, 200032 China; 6 Institute of Neuroscience, State Key Laboratory of Neuroscience, CAS Center for Excellence in Brain Science and Intelligence Technology, Chinese Academy of Sciences, Shanghai, 200031 China

**Keywords:** Alzheimer’s disease, Brain glucose metabolism, Thiamine diphosphate, Amyloid-β, Position emission tomography

## Abstract

**Background:**

The underlying mechanism of brain glucose hypometabolism, an invariant neurodegenerative feature that tightly correlates with cognitive impairment and disease progression of Alzheimer’s disease (AD), remains elusive.

**Methods:**

Positron emission tomography with 2-[^18^F]fluoro-2-deoxy-d-glucose (FDG-PET) was used to evaluate brain glucose metabolism, presented as the rate of 2-[^18^F]fluoro-2-deoxy-d-glucose standardized uptake value ratio (FDG SUVR) in patients with AD or control subjects and in mice with or without thiamine deficiency induced by a thiamine-deprived diet. Brain amyloid-β (Aβ) deposition in patients with clinically diagnosed AD was quantified by performing assays using ^11^C-Pittsburgh compound B PET. The levels of thiamine metabolites in blood samples of patients with AD and control subjects, as well as in blood and brain samples of mice, were detected by high-performance liquid chromatography with fluorescence detection.

**Results:**

FDG SUVRs in frontal, temporal, and parietal cortices of patients with AD were closely correlated with the levels of blood thiamine diphosphate (TDP) and cognitive abilities, but not with brain Aβ deposition. Mice on a thiamine-deprived diet manifested a significant decline of FDG SUVRs in multiple brain regions as compared with those in control mice, with magnitudes highly correlating with both brain and blood TDP levels. There were no significant differences in the changes of FDG SUVRs in observed brain regions between amyloid precursor protein/presenilin-1 and wild-type mice following thiamine deficiency.

**Conclusions:**

We demonstrate, for the first time to our knowledge, in vivo that TDP reduction strongly correlates with brain glucose hypometabolism, whereas amyloid deposition does not. Our study provides new insight into the pathogenesis and therapeutic strategy for AD.

**Electronic supplementary material:**

The online version of this article (10.1186/s13195-018-0354-2) contains supplementary material, which is available to authorized users.

## Background

Alzheimer’s disease (AD) is the most prevalent type of dementia and a devastating burden for individuals and their families [1]. A main hypothesis in the field is that AD consists of a sequence of pathological process, with β-amyloid (Aβ) accumulation being the initial causal event (the “amyloid cascade hypothesis”) [2, 3], subsequently and progressively replaced by more complex mechanisms [4–6]. However, recent imaging studies using positron emission tomography (PET) showed that neuronal injury biomarkers and tau pathology can occur independently of Aβ deposition [7–11]. Especially after the onset of clinical symptoms, amyloid deposition and cognitive dysfunction appear to become decoupled. We thus surmise that other mechanisms may contribute to AD cognitive dysfunction. A better understanding of the mechanisms that drive AD clinical deterioration and definitively connect progressive cognitive deterioration to biomarkers would yield important insights into the disease’s pathogenesis. This has direct implications for new strategies for diagnosis of and therapy for the disease.

Brain glucose hypometabolism is an invariant feature and neurodegenerative index of AD, preceding the onset of overt clinical symptoms by decades [12, 13]. The reduction in cerebral glucose metabolism correlates closely with the degree of cognitive impairment and the disease progression of AD, reflecting the extent of synaptic dysfunction and neurodegeneration [14]. Disrupted cerebral glucose metabolism has been proposed to mediate Aβ deposition and tau hyperphosphorylation by inducing oxidative stress, inflammation, mitochondrial dysfunction, autophagy impairment, excitotoxicity, and apoptosis [4]. The improvement in brain glucose metabolism by intranasal insulin administration significantly ameliorates impaired cognitive abilities in patients with AD, including delayed memory and verbal function [15, 16]. Thus, brain glucose hypometabolism may directly contribute to cognitive impairment and neurodegeneration in AD. To identify the mechanisms underlying brain glucose hypometabolism in AD would help further the understanding of AD pathogenesis and identify new therapeutic targets.

Reduction of thiamine diphosphate (TDP) levels and the activities of TDP-dependent key enzymes in glucose metabolism has been reported in blood samples and autopsied brain samples of patients with AD [17–21]. Thus, we hypothesized that TDP reduction contributes to cerebral glucose hypometabolism in AD. To test this hypothesis, we investigated whether the level of blood thiamine metabolites correlates with brain glucose metabolism as evaluated by positron emission tomography with 2-[^18^F]fluoro-2-deoxy-d-glucose (FDG-PET) and cerebral amyloid deposition as assayed by PET with ^11^C-Pittsburgh compound B (PiB-PET) in patients with AD. Wild-type, thiamine-deficient, and amyloid precursor protein/presenilin-1 (APP/PS1) transgenic mice were also used to further examine the findings derived from clinical investigation.

## Methods

### Study and subjects

This study was approved by the Committee of Medical Ethics of Zhongshan Hospital, Fudan University. All subjects were volunteers recruited from Zhongshan Hospital, Fudan University, Shanghai, China, and from Daping Hospital, Third Military Medical University, Chongqing, China. All patients had cranial magnetic resonance imaging and/or computed tomographic (CT) scans and were diagnosed by neurologists specializing in dementia (Drs. Chunjiu Zhong, Yanjiang Wang, Guoqiang Fei, and Lirong Jin) according to the *Diagnostic and Statistical Manual of Mental Disorders, Fourth Edition,* and diagnostic guidelines for AD (National Institute on Aging-Alzheimer’s Association workgroups) [22]. All patients (and/or their caregivers) underwent neurological and neuropsychological evaluations, including the Mini Mental State Examination (MMSE), Clinical Dementia Rating (CDR), activities of daily living, and Hamilton Rating Scale for Depression. Blood folate, vitamin B_12_, and thyroid function were also measured in patients with AD. The following subjects were excluded from this study: (1) subjects with chronic alcohol abuse, (2) subjects with disorders of the gastrointestinal tracts, (3) subjects taking thiamine supplements within the past month, (4) patients with major depression, and (5) patients with thyroid dysfunction or reduced levels of blood folate and vitamin B_12_. Informed consent was obtained from all participating subjects. The demographic data of the patients are listed in Table 1.

**Table 1:** Characteristics of study subjects

Characteristics	Control (*n* = 14)	AD for TDP and FDG-PET (*n* = 14)	AD for TDP and PiB-PET (*n* = 35)	AD for FDG and PiB-PET (*n* = 23)
Male sex, *n* (%)	6 (42.9)	6 (42.9)	20 (47.1%)	14 (60.9%)
Age, years	68.1 ± 2.9	69.3 ± 2.7	69.4 ± 10.9	68.6 ± 7.83
Education level, years	13.4 ± 0.6	7.5 ± 1.2^a^	9.9 ± 1.46	8.3 ± 0.75
MMSE score	29.1 ± 0.2 (26-30)	16.6 ± 1.4 (6-24)^a^	17.6 ± 7.0 ^a^	15.3 ± 8.14^a^
APOE ε4, *n* (%)	3 (23.1%)^b^	10 (71.4%)^a^	19 (54.3%)^a^	10 (43.5%)^a^
Folate, ng/ml	–	8.8 ± 0.7	9.72 ± 0.90	22.9 ± 3.12
Vitamin B_12_, pg/ml	–	499.6 ± 59.1	489.2 ± 47.2	329.4 ± 77.5
TDP, nmol/L	117.1 ± 4.7	84.9 ± 7.1^a^	101.3 ± 30.3^a^	–
SUVR of FDG or PiB in frontal cortex	1.25 ± 0.030	1.10 ± 0.055^a^	1.9 ± 0.5	1.04 ± 0.02^a^1.9 ± 0.5
SUVR of FDG or PiB in temporal cortex	1.17 ± 0.028	0.95 ± 0.051^a^	1.7 ± 0.5	1.05 ± 0.02^a^2.00 ± 0.08
SUVR of FDG or PiB in parietal cortex	1.16 ± 0.031	0.97 ± 0.044^a^	1.7 ± 0.4	0.92 ± 0.02^a^1.96 ± 0.07

*Abbreviations: AD* Alzheimer’s disease, *APOE* Apolipoprotein E, *FDG* 2-[^18^F]fluoro-2-deoxy-d-glucose, *MMSE* Mini Mental State Examination, *PET* Positron emission tomography, *PiB* ^11^C-Pittsburgh compound B, *SUVR* 2-[^18^F]fluoro-2-deoxy-d-glucose standardized uptake value ratio, *TDP* Thiamine diphosphate

Data are presented as mean ± SEM or count (percent)

^a^*P* < 0.001 vs. control

^b^Unable to determine for one subject

### Animals

All animal care and experimental procedures were approved by the Medical Experimental Animal Administrative Committee of Fudan University and by the Institutional Animal Care and Use Committee of the Institute of Neuroscience, Shanghai Institutes for Biological Sciences, Chinese Academy of Sciences. APP/PS1 (strain 4462) transgenic mice were purchased from The Jackson Laboratory (Bar Harbor, ME, USA) and used as heterozygotes. APP/PS1 and C57BL/6 mice were also used for thiamine deficiency (TD) experiments. All animals were housed in a humidity- and temperature-controlled environment with 12-h/12-h light/dark cycles and free access to food and water. TD in mice was induced by feeding them a thiamine-depleted diet (Bio-Serv, Flemington, NJ, USA) and distilled water ad libitum for up to 26 consecutive days. Control animals received a thiamine-containing diet (Bio-Serv) and distilled water ad libitum.

### Positron emission tomography

Approximately 10 mCi ^18^F-FDG or ^11^C-PiB was injected through the opisthenar vein within 60 seconds. From 0 to 60 minutes after injection, 3D dynamic PET acquisition was performed using a Biograph 44 TruePoint PET•CT scanner (Siemens Medical Solutions, Malvern, PA, USA) and a Discovery ST system (GE Healthcare, Wauwatosa, WI, USA) for FDG-PET and a Biograph 64 TruePoint PET•CT scanner for PiB-PET. CT scanning was also performed for attenuation correction, and coregistered images were displayed on a workstation. The 40- to 60-minute static images were reconstructed using an iterative 3D method with a Gaussian filter (6 mm FWHM). The pixel size was 2.0 mm, and the slice thickness was 1.5 mm.

FDG-PET images were coregistered to the automated anatomic labeling brain template by statistical parametric mapping (SPM8; Wellcome Trust Centre for Neuroimaging, Department of Cognitive Neurology, London, UK), and 116 regions of interest (ROIs) were automatically extracted as described in a previous study [23]. FDG uptake reflects the status of brain glucose metabolism and was represented by the standard uptake value ratios (SUVRs), which were defined as the ratio of SUV in the ROI to that in the cerebellar cortex. The mean and maximum FDG uptake values were calculated using PMOD v3.3 software (PMOD Technologies Ltd., Zurich, Switzerland).

The deposition/retention of ^11^C-PiB was calculated as previously described [24]. Seven ROIs were studied per subject, including the frontal cortex, temporal cortex, parietal cortex, hippocampus, anterior cingulate cortex, posterior cingulate cortex, and cerebellar cortex. The ROI placement procedure was performed as described previously. PiB-PET images were analyzed by two independent experienced physicians who were blinded to the clinical data. The average of the two independent SUVRs was then calculated.

### Micro-PET scan

The radiolabeling synthesis of ^18^F-FDG and PET-CT scanning was conducted as described previously [23, 25]. Before ^18^F-FDG injection, mice were fasted overnight with free access to water. Mice were injected with approximately 18.5 MBq (500 μCi) of ^18^F-FDG through the tail vein. Thirty minutes later, mice were anesthetized with isoflurane, and PET-CT scanning was performed, which provided a 12.7-cm axial field of view and an average intrinsic spatial resolution of 1.75 mm. The entire static process included CT scanning for 10 minutes and PET scanning for 20 minutes. PET images were reconstructed using a Fourier rebinning and 2D filtered back-projection method (ramp filter and cutoff at Nyquist frequency) with an image matrix of 128 × 128 × 159, resulting in a pixel size of 0.77 mm and a slice thickness of 0.796 mm on 3D images. The images were analyzed using Inveon Research Workplace software (Siemens Medical Solutions). ROIs were automatically extracted from all micro-PET images using ^18^F-FDG murine brain templates of the Inveon Research Workplace software. Relative FDG uptake was calculated for each ROI using the average tissue activity in the region.

### Measurement of TDP, thiamine monophosphate, and thiamine

Human and murine fresh whole blood was collected, anticoagulated with heparin, and deproteinized with 7.2% or 7.6% perchloric acid, respectively. Murine brain tissue was homogenized with 100 mM K_2_HPO_4_ (pH 5.0) and deproteinized with isometric 7.2% perchloric acid. All samples were centrifuged, and supernatants were collected. The levels of TDP, thiamine monophosphate (TMP), and thiamine were measured as previously described [26].

### Immunohistochemical staining

Mice were deeply anesthetized with 0.14 g/kg sodium pentobarbital, intracardially perfused with PBS, and fixed using 4% paraformaldehyde. Serial coronal sections (35 μm) were cut with a sliding microtome (Leica Biosystems, Buffalo Grove, IL, USA) and stained as freely floating sections. For examination of amyloid plaques, sections were incubated in 88% formic acid at room temperature for 8 minutes. After a washing step in PBS at pH 7.4, sections were blocked in PBS containing 5% bovine serum albumin and 0.5% Triton X-100 for 2 h at 37 °C, then incubated with mouse anti-Aβ antibody (1:1000, Covance 6E10; BioLegend, San Diego, CA, USA) overnight at 4 °C. After further PBS washing, sections were incubated with goat antimouse antibody conjugated to Alexa Fluor 488 (1:500; Thermo Fisher Scientific, Waltham, MA, USA) for 2 h at 37 °C and mounted on (3-aminopropyl)triethoxysilane-coated glass slides. Z-stack images were taken using a Zeiss Pascal (Carl Zeiss Microscopy, Jena, Germany) or Nikon A1 (Nikon Instruments, Tokyo, Japan) laser scanning microscope with a ×20 lens objective. Results were quantified using Image-Pro Plus (Media Cybernetics, Rockville, MD, USA).

### Enzyme-linked immunosorbent assay for β-amyloid 1–42 and β-amyloid 1–40

The levels of Aβ 1–42 (Aβ_42_) and Aβ 1–40 (Aβ_40_) were determined by enzyme-linked immunosorbent assay (Human Aβ42 or Aβ40 Colorimetric ELISA; Thermo Fisher Scientific, Grand Island, NY, USA) according to the manufacturer’s instructions. Final values of Aβ were expressed as nanograms per gram of brain tissue (wet weight).

### Statistical analysis

PASW Statistics software (version 18.0; SPSS Inc., Chicago, IL, USA) and Prism 6 (version 6.01; GraphPad Software, La Jolla, CA, USA) were used for statistical analyses. Student’s *t* test, one-way analysis of variance (ANOVA), or chi-square tests were used to compare demographic data. Two-way ANOVA with Tukey’s multiple comparisons posttest was used to compare TDP, TMP, thiamine levels, and SUVRs based on FDG-PET. Linear regression analysis was conducted to yield the Pearson product-moment correlation coefficient (*r*) for identifying the correlation between the levels of blood thiamine metabolites and FDG SUVRs or PiB retention, as well as the correlation between FDG SUVRs and PiB retention. Student’s *t* test for single comparisons or one-way ANOVA for multiple comparisons with appropriate Tukey’s or Dunnett’s multiple comparisons tests were used to identify statistical differences. Results were represented as the mean ± SEM. All conditions statistically different from their control are indicated by asterisks in the figures.

## Results

### TDP reduction correlates significantly with brain glucose hypometabolism

**Fig. 1:**
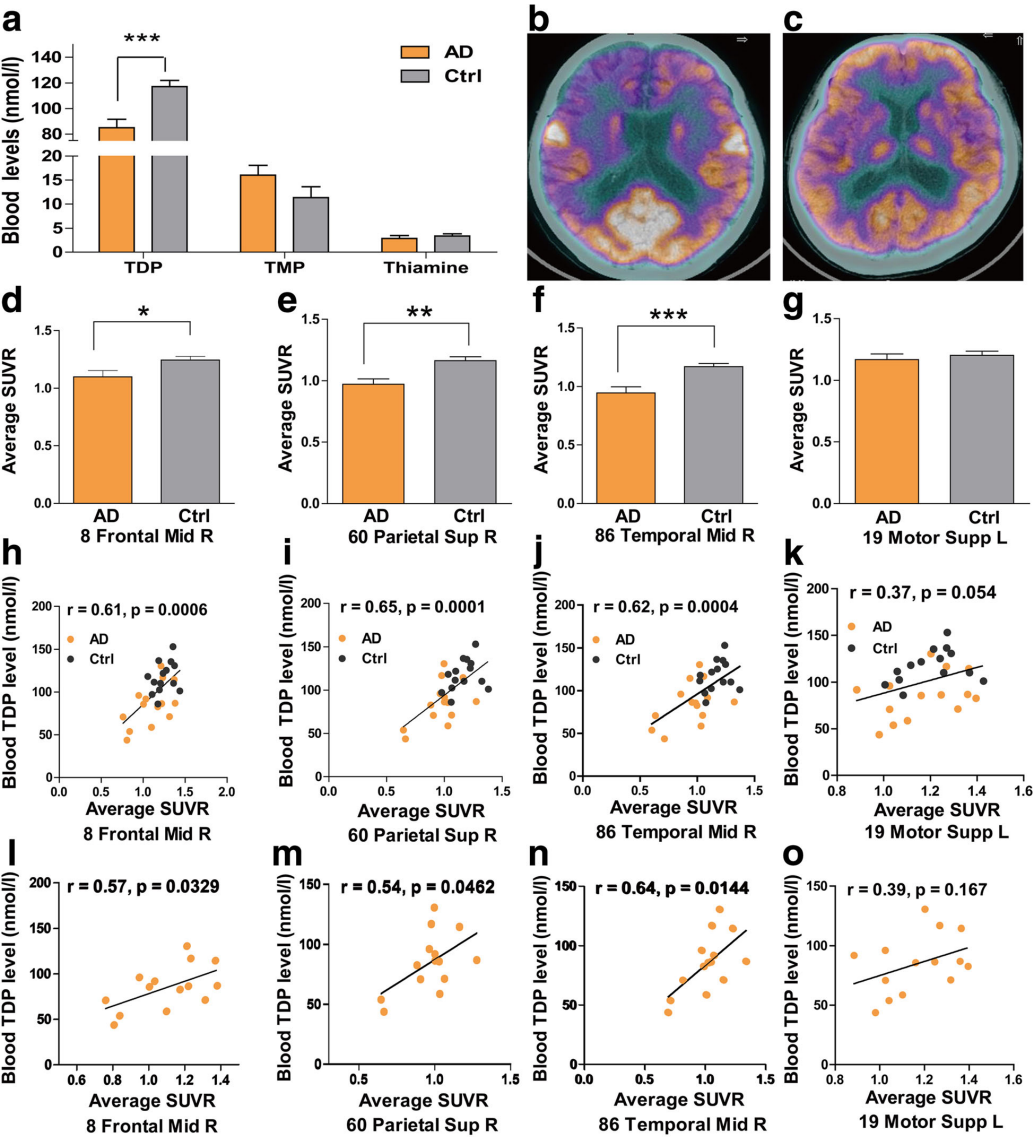
Blood thiamine diphosphate (TDP) levels correlate with brain glucose metabolism in patients with Alzheimer’s disease (AD). **a** Blood TDP levels were reduced, whereas thiamine monophosphate (TMP) and thiamine levels were not significantly altered in patients with AD as compared with those in control (Ctrl) subjects (TDP 85.04 ± 6.59 vs. 117.23 ± 4.83 nmol/L; TMP 16.02 ± 2.04 vs. 11.34 ± 2.27 nmol/L; thiamine 2.85 ± 0.62 vs. 3.37 ± 0.98 nmol/L, *n* = 14). **b**, **c** Representative Positron emission tomography with 2-[^18^F]fluoro-2-deoxy-d-glucose (FDG-PET) images of patient with AD (**b**) and control subject (**c**). **d–g** The standardized uptake value ratios (SUVRs) of representative brain regions in patients with AD were significantly reduced as compared with those in control subjects (**d** 1.10 ± 0.055 vs. 1.25 ± 0.030 in right midfrontal lobe; **e** 0.97 ± 0.044 vs. 1.16 ± 0.031 in right superior parietal lobe; **f** 0.95 ± 0.051 vs. 1.17 ± 0.028 in right midtemporal lobe; **g** 1.17 ± 0.044 vs. 1.20 ± 0.033 in left motor lobe; *n* = 14). **h–k** Correlations between SUVRs and blood TDP levels of representative brain regions in combined group. ***l*–o** Correlations between SUVRs and blood TDP levels of representative brain regions in patients with AD. *R* Right. * *P* < 0.05, ** *P* < 0.01, and *** *P* < 0.001

Blood TDP levels in patients with AD were significantly reduced as compared with levels in age- and sex-matched control subjects (*P* < 0.001, *n* = 14) (Fig. 1a). This result was consistent with that of our previous study [21]. Glucose metabolism presented as SUVR was significantly lower in patients with AD than in control subjects in representative brain regions, including the frontal cortex, parietal cortex, and temporal cortex (*P* < 0.05, 0.01, or 0.001, respectively) (Fig. 1b–f), but not in the motor cortex (*P* > 0.05) (Fig. 1g). Glucose metabolism in representative brain regions positively correlated with blood TDP levels in both patients with AD (*r* = 0.57, *P* < 0.05 for frontal cortex; *r* = 0.54, *P* < 0.05 for parietal cortex; *r* = 0.64, *P* < 0.05 for temporal cortex; *n* = 14) (Fig. 1*l*–n) and the combined group (*r* = 0.61, *P* < 0.001 for frontal cortex; *r* = 0.65, *P* < 0.001 for parietal cortex; *r* = 0.62, *P* < 0.001 for temporal cortex; *n* = 28) (Fig. 1h–j), but not in control subjects (*see* Additional file 1: Figure S1) or with blood TMP and thiamine levels in any group (*see* Additional file 1: Figure S2). As a control, there were no significant correlations between blood TDP level and glucose metabolism in the supplementary motor cortex of both patients with AD (Fig. 1o) and the combined group (Fig. 1k).

**Fig. 2:**
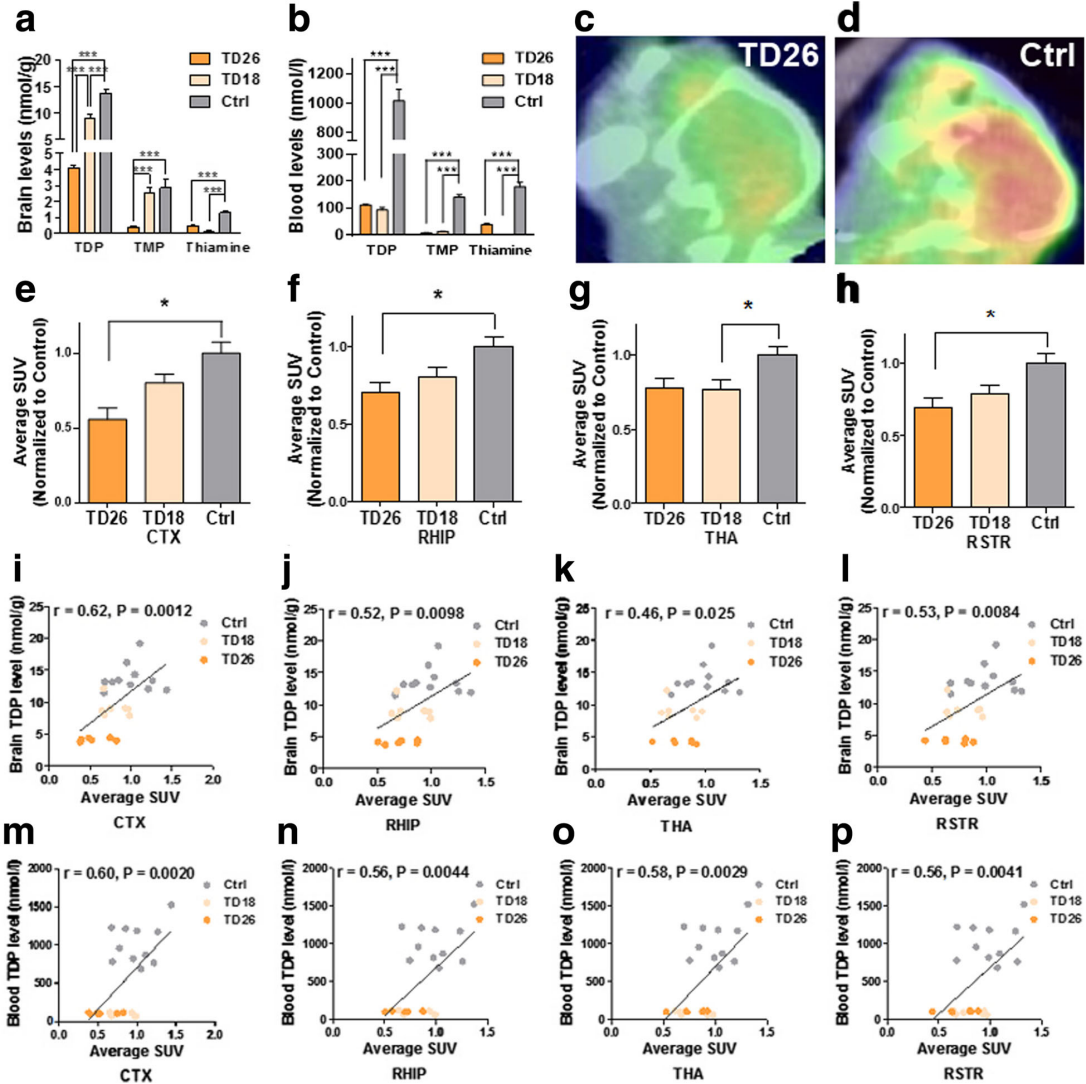
Thiamine diphosphate (TDP) reduction significantly impairs brain glucose metabolism. **a** Brain TDP, thiamine monophosphate (TMP), and thiamine levels in mice with thiamine deficiency on the 26th day (TD26) and the 18th day (TD18) and in control mice (TDP 4.13 ± 0.11, 9.10 ± 0.53 vs. 13.70 ± 0.67 nmol/g; TMP 0.39 ± 0.03, 2.54 ± 0.30 vs. 2.84 ± 0.52 nmol/g; thiamine 0.46 ± 0.05, 0.13 ± 0.01 vs. 1.32 ± 0.01 nmol/g; two-way analysis of variance [ANOVA] with Tukey’s multiple comparisons posttest; *n* = 6, 7, and 11, respectively). **b** Blood TDP, TMP, and thiamine in TD26, TD18, and control mice (TDP 111.64 ± 2.39, 93.72 ± 8.83 vs. 1019.08 ± 78.80 nmol/L; TMP 7.83 ± 1.10, 12.93 ± 1.08 vs. 140.99 ± 9.31 nmol/L; thiamine 38.94 ± 4.68, 2.34 ± 0.22 vs. 179.54 ± 17.14 nmol/L; two-way ANOVA with Tukey’s multiple comparisons posttest; *n* = 6, 7, and 11, respectively). **c**, **d** Representative micro-positron emission tomography with 2-[^18^F]fluoro-2-deoxy-d-glucose (FDG-PET) images of (**c**) TD26 and (**d**) control mice. **e**–**h** FDG uptake of representative brain regions represented by standardized uptake value ratios (SUVRs) normalized to control mice in TD26 mice and TD18 mice was significantly reduced as compared with that in control mice (cortex [CTX] 0.56 ± 0.08, 0.80 ± 0.06 vs. 1.00 ± 0.07, *F* = 8.78, *P* < 0.01; right hippocampus [RHIP] 0.71 ± 0.06, 0.80 ± 0.06 vs. 1.00 ± 0.07, *F* = 5.28, *P* < 0.05; thalamus [THA] 0.78 ± 0.06, 0.77 ± 0.06 vs. 1.00 ± 0.06, *F* = 5.08, *P* < 0.05; right striatum [RSTR] 0.70 ± 0.07, 0.79 ± 0.06 vs. 1.00 ± 0.07, *F* = 5.57, *P* < 0.05; one-way ANOVA with Tukey’s multiple comparisons posttest; *n* = 6, 7, and 11, respectively). **i**–***l*** Correlations between brain TDP levels and SUVRs in representative brain regions of mice (*n* = 24). **m**–**p** Correlations between blood TDP levels and SUVRs in representative brain regions of mice (*n* = 24). * *P* < 0.05, ** *P* < 0.01, and *** *P* < 0.001

To investigate whether TDP reduction directly results in brain glucose hypometabolism, mice were fed a thiamine-deprived diet and assayed for glucose metabolism in representative brain regions using micro-FDG-PET on either the 18th (TD18) or 26th day (TD 26) after diet initiation. At both time points, the levels of thiamine metabolites were significantly reduced in both brain and blood samples (all *P* < 0.001, *n* = 11 for control, *n* = 7 for TD18, *n* = 6 for TD26) (Fig. 2a, b). Also at both time points, TD mice showed significant reduction of glucose metabolism as compared with control mice in brain regions including cortex (*F* = 8.78, *P* < 0.01), hippocampus (*F* = 5.28, *P* < 0.05), thalamus (*F* = 5.08, *P* < 0.05), and striatum (*F* = 5.57, *P* < 0.05) (Fig. 2c–h). Furthermore, glucose metabolism in representative brain regions positively correlated with brain and blood TDP levels, including the cortex (*r* = 0.62, *P* < 0.01 for brain TDP level; *r* = 0.60, *P* < 0.01 for blood TDP level), hippocampus (*r* = 0.52, *P* < 0.01 for brain TDP level; *r* = 0.56, *P* < 0.01 for blood TDP level), thalamus (*r* = 0.46, *P* < 0.05 for brain TDP level; *r* = 0.58, *P* < 0.01 for blood TDP level), and striatum (*r* = 0.53, *P* < 0.01 for brain TDP level; *r* = 0.55, *P* < 0.01 for blood TDP level; *n* = 24) (Fig. 2i–p), but not in control mice (*see* Additional file 1: Figure S3).

### Brain amyloid deposition did not significantly affect TDP level

**Fig. 3:**
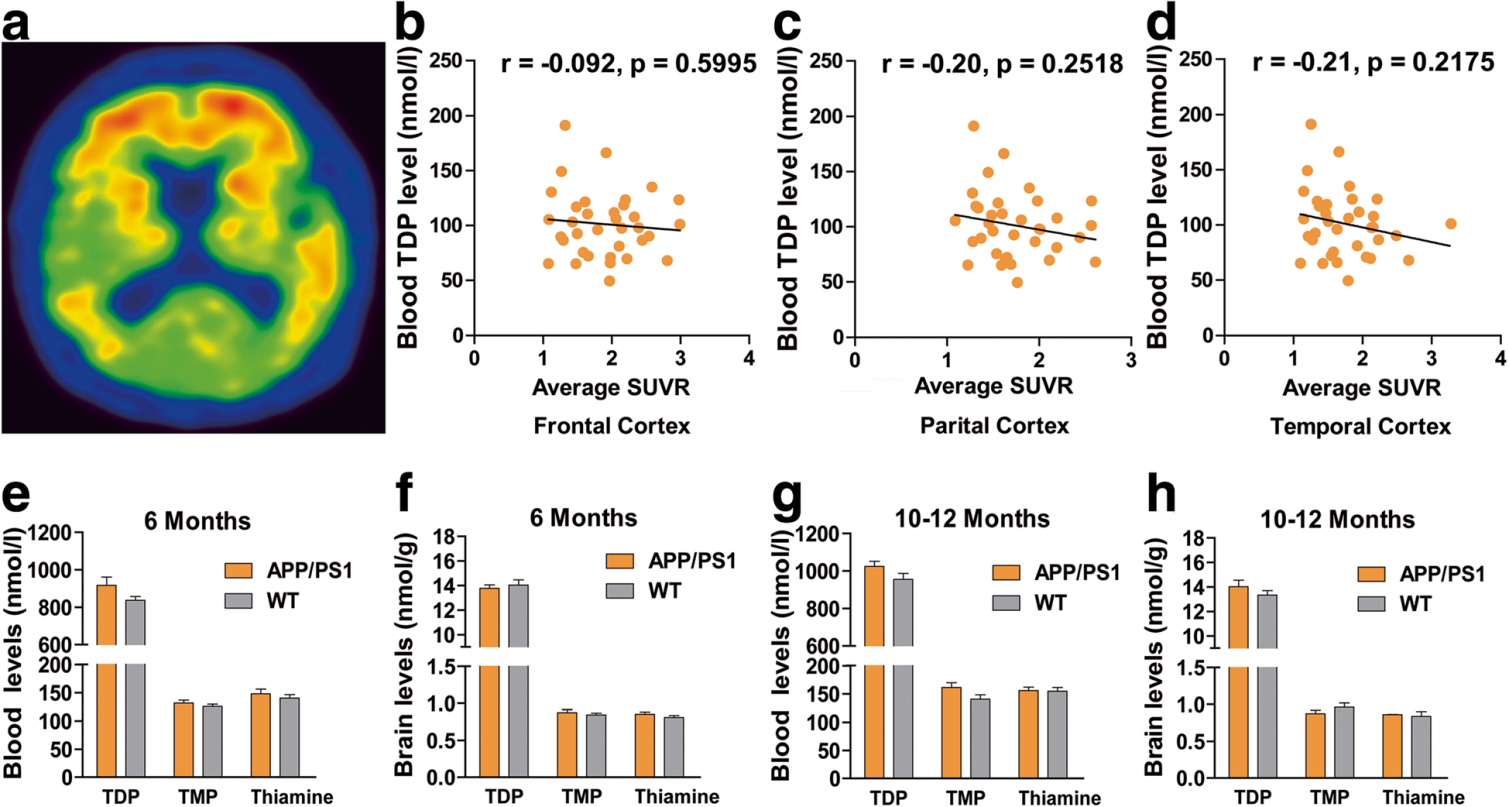
Brain amyloid deposition does not significantly correlate with thiamine diphosphate (TDP) level. **a** Representative image of positive scan obtained by positron emission tomography with ^11^C-Pittsburgh compound B in patients with Alzheimer’s disease (AD). **b–d** Blood TDP levels did not closely correlate with average standardized uptake value ratios (SUVRs) of representative brain regions in patients with AD (*P* > 0.05; *n* = 35). **e–h** Comparison of the levels of blood (**e**, **g**) and brain (**f**, **h**) thiamine metabolites between 6-month-old (**e**, **f**) or 10- to 12-month old (**g**, **h**) amyloid precursor protein/presenilin-1 (APP/PS1) transgenic and age-matched wild-type (WT) mice (all *P* > 0.05; *n* = 12 for each group)

There were no significant correlations between blood TDP levels and amyloid deposition in representative brain regions of patients with AD as evaluated by PiB-PET scanning (*r* = −0.092, *P* > 0.05 for frontal cortex; *r* = −0.20, *P* > 0.05 for parietal cortex; *r* = −0.21, *P* > 0.05 for temporal cortex; *n* = 35) (Fig. 3a–d). Also, neither blood TMP nor thiamine levels significantly correlated with brain amyloid accumulation (*see* Additional file 1: Figure S4). Furthermore, the levels of blood and brain TDP, TMP, and thiamine were not significantly altered in APP/PS1 transgenic mice as compared with those in age-matched wild-type mice (all *P* > 0.05 for 6- and 10- to 12-month-old mice; *n* = 12) (Fig. 3e–h).

### Amyloid deposition does not enhance TD-induced brain glucose hypometabolism

**Fig. 4:**
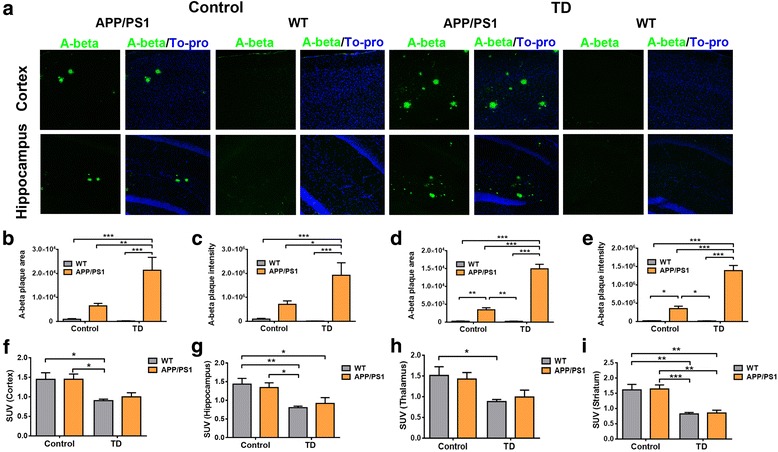
Amyloid deposition does not enhance the vulnerability of brain glucose hypometabolism. **a** Representative cortical or hippocampal slices of 6-month-old amyloid precursor protein/presenilin-1 (APP/PS1) transgenic and wild-type (WT) mice following thiamine deficiency (TD) or control diet with immunostaining for β-amyloid (Aβ; *green*) and TO-PRO (*blue*; Thermo Fisher Scientific). **b–e** The area and intensity of amyloid plaques in cortex and hippocampus were significantly elevated in APP/PS1 transgenic mice as compared with age-matched WT mice. Thiamine diphosphate (TDP) reduction significantly enhanced the area and intensity of amyloid plaques in cortex and hippocampus of 6-month-old APP/PS1 transgenic mice (*P* < 0.001, *P* < 0.01, or *P* < 0.05, respectively; two-way analysis of variance (ANOVA) with Tukey’s multiple comparisons posttest; *n* = 5 or 6). **f–i** TDP reduction induced a similar reduction of glucose metabolism in representative brain regions in APP/PS1 transgenic and WT mice. There were no significant interactions between TDP reduction and amyloid plaques (*F*_1,19_ = 0.15, *P* > 0.05 for cortex; *F*_1,19_ = 0.66, *P* > 0.05 for hippocampus; *F*_1,19_ = 0.39, *P* > 0.05 for thalamus; *F*_1,19_ = 0.00, *P* > 0.05 for striatum; *P* < 0.001, *P* < 0.01, or *P* < 0.05, respectively; two-way ANOVA with Tukey’s multiple comparisons posttest; *n* = 5 or 6). SUV Standardized uptake value. *P < 0.05, **P < 0.01, and ***P < 0.001

Previous studies suggested that TD exacerbated the plaque pathology in APP/PS1 mice [27–29]. We assumed that brain glucose hypometabolism induced by TD was vulnerable under the circumstance of exacerbated amyloid deposition. Consistent with the results of previous studies [27–29], TD significantly increased the number and average area of amyloid plaques in the cortex and hippocampus of 6-month-old APP/PS1 transgenic mice (*P* < 0.001, *P* < 0.01, or *P* < 0.05, respectively; *n* = 5 or 6) (Fig. 4a–e). However, there were no significant differences in the degrees of brain glucose hypometabolism induced by TD in representative brain regions between APP/PS1 mice and wild-type mice (interaction *F*_1,19_ = 0.15, *P* > 0.05 for cortex; interaction *F*_1,19_ = 0.66, *P* > 0.05 for hippocampus; interaction *F*_1,19_ = 0.39, *P* > 0.05 for thalamus; interaction *F*_1,19_ = 0.00, *P* > 0.05 for striatum; *n* = 5 or 6) (Fig. 4f–i). In other words, amyloid deposition did not aggravate TD-induced brain glucose hypometabolism.

### Brain amyloid load and glucose metabolism are not correlated in both patients with AD and APP/PS1 transgenic mice

**Fig. 5:**
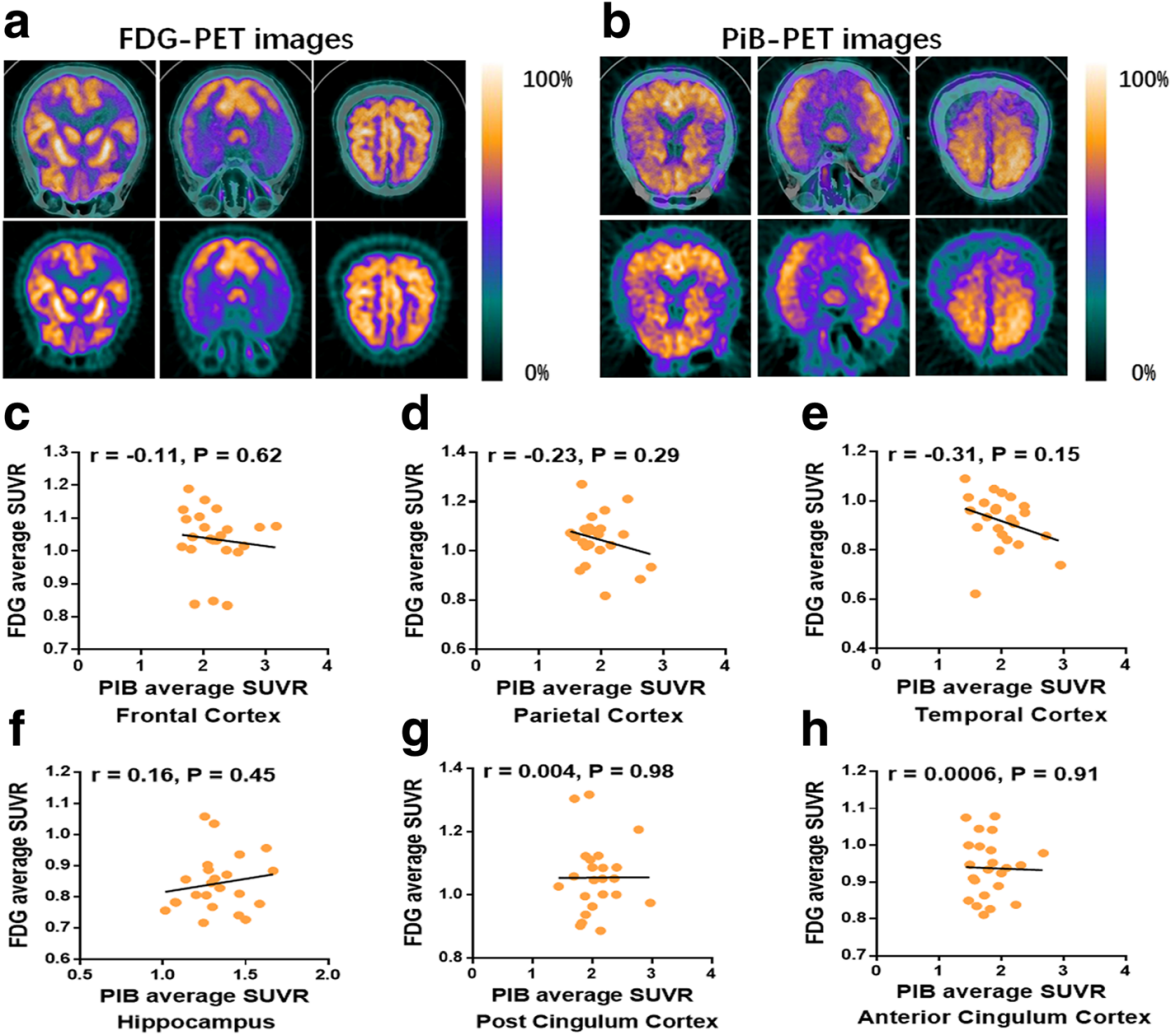
No correlation between brain amyloid load and glucose metabolism in patients with Alzheimer’s disease (AD). **a**, **b** Representative 2-[^18^F]fluoro-2-deoxy-d-glucose positron emission tomography-computed tomographic (FDG-PET-CT) scan (**a**) and ^11^C-Pittsburgh compound B (PiB)-PET-CT scan (**b**) in the same patient with AD. **c–h** Correlations between standardized uptake value ratios (SUVRs) of FDG-PET scans and SUVRs of PiB-PET scans of representative brain regions in patients with AD. No significant correlations were found (*n* = 23, all *P* > 0.05)

We further assayed the relationship between brain Aβ deposition and glucose metabolism in patients with AD and APP/PS1 transgenic mice. Patients with AD were simultaneously examined using PiB-PET and FDG-PET within a 1-week time window. Consistent with previous studies [7, 8], we found that there were no significant correlations between brain glucose metabolism and brain amyloid load in various brain regions of patients with AD, including frontal cortex (*r* = −0.11, *P* > 0.05), parietal cortex (*r* = −0.23, *P* > 0.05), temporal cortex (*r* = −0.31, *P* > 0.05), hippocampus (*r* = 0.16, *P* > 0.05), posterior cingulate cortex (*r* = 0.004, *P* > 0.05), and anterior cingulate cortex (*r* = 0.006, *P* > 0.05; *n* = 23) (Fig. 5a–h). Brain glucose metabolism correlated closely with the degree of cognitive impairment assayed by the MMSE score, activities of daily living, and CDR, but not brain amyloid deposition, in patients with AD (Table 2).

**Table 2:** Brain glucose metabolism correlated with measurement instrument results but not brain amyloid load in patients with Alzheimer’s disease

	Frontal cortex		Parietal cortex		Temporal cortex		Hippocampus		Posterior cingulate cortex		Anterior cingulate cortex	
Correlations	*r* Value	*P* value	*r* Value	*P* value	*r* Value	*P* value	*r* Value	*P* value	*r* Value	*P* value	*r* Value	*P* value
FDG SUVR and MMSE	0.47	0.02^a^	0.45	0.03^a^	0.62	0.001^a^	0.35	0.10	0.36	0.09	0.22	0.31
PiB SUVR and MMSE	−0.28	0.19	−0.08	0.71	−0.34	0.12	−0.07	0.74	−0.004	0.87	−0.17	0.45
FDG SUVR and ADL	−0.65	0.008^a^	−0.40	0.06	−0.56	0.006^a^	−0.39	0.07	−0.46	0.03^a^	−0.08	0.72
PiB SUVR and ADL	0.03	0.43	0.01	0.95	0.22	0.30	0.05	0.82	0.02	0.56	0.05	0.82
FDG SUVR and CDR	−0.46	0.03^a^	−0.12	0.60	−0.43	0.04^a^	−0.24	0.28	−0.22	0.32	−0.06	0.79
PiB SUVR and CDR	0.14	0.53	−0.01	0.95	0.17	0.44	−0.12	0.57	−0.21	0.34	0.05	0.83

^a^*P* < 0.05, *P* < 0.01 or *P* < 0.001

*Abbreviations: ADL* Activities of daily living, *CDR* Clinical Dementia Rating, *FDG* 2-[^18^F]fluoro-2-deoxy-d-glucose, *MMSE* Mini Mental State Examination, *PiB* ^11^C-Pittsburgh compound B, *SUVR* Standardized uptake value ratio

**Fig. 6:**
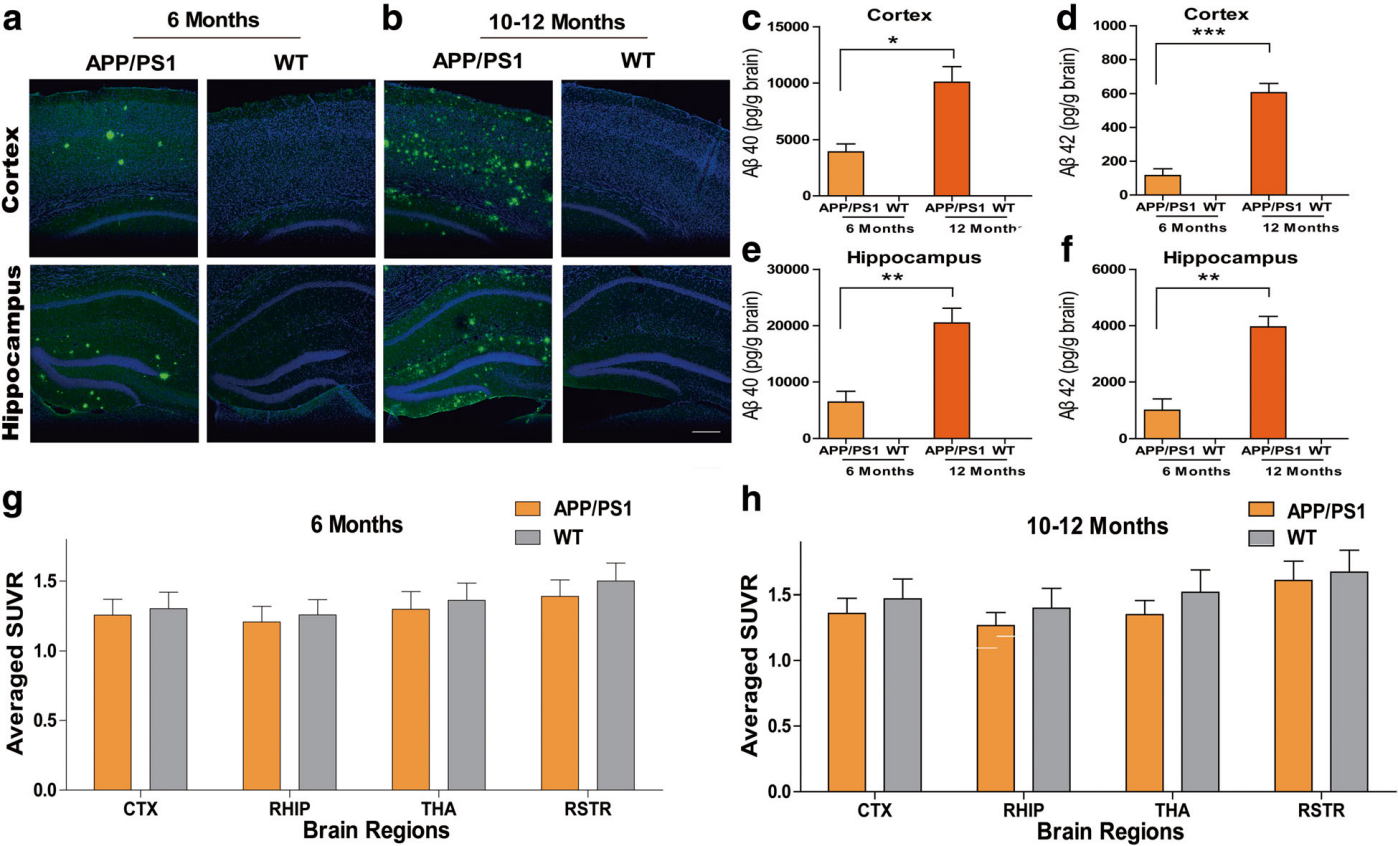
Brain amyloid load did not affect glucose metabolism in amyloid precursor protein/presenilin-1 (APP/PS1) mice. **a**, **b** Representative brain slices of APP/PS1 transgenic and age-matched wild-type (WT) mice were immunostained for β-amyloid (Aβ; *green*) and TO-PRO (*blue*; Thermo Fisher Scientific). **c–f** Cortical and hippocampal Aβ 1–42 (Aβ_42_) and Aβ 1–40 (Aβ_40_) levels measured by enzyme-linked immunosorbent assay were significantly elevated in APP/PS1 mice as compared with those in age-matched WT mice (APP/PS1 transgenic mice at age 6 months or 10–12 months, *n* = 3 or 4; age-matched WT mice, *n* = 5). **g**, **h** 2-[^18^F]fluoro-2-deoxy-d-glucose standardized uptake value ratios (SUVRs) in different brain regions of 6-month-old (**g**) and 12-month-old (**h**) APP/PS1 transgenic mice and age-matched controls. No significant differences were found. (*n* = 10 for both groups at 6 months; *n* = 8 for APP/PS1 mice and *n* = 9 for WT mice at 10–12 months). *CTX* Cortex, *RHIP* Right hippocampus, *RSTR* Right striatum, *THA* Thalamus. *P < 0.05, **P < 0.01, and ***P < 0.001

APP/PS1 transgenic mice (6 or 10–12 months old) that exhibited significantly elevated brain amyloid plaques and soluble Aβ levels (*P* < 0.05, *P* < 0.01 and *P* < 0.001, respectively; *n* = 3–5) (Fig. 6a–f) were used to further investigate the relationship between brain amyloid accumulation and brain glucose metabolism. FDG uptakes in representative brain regions of APP/PS1 transgenic mice in both groups were not significantly altered as compared with those in age-matched wild-type mice (all *P* > 0.05; *n* = 8–10) (Fig. 6g, h).

## Discussion

TDP is well known as a critical coenzyme closely associated with glucose metabolism. We demonstrate, for the first time in vivo, to our knowledge, the relationship between TDP level and brain glucose metabolism. The results show that TDP reduction significantly impaired brain glucose metabolism in mice, manifested as significant declines of FDG SUVRs in multiple brain regions of TD mice as compared with FDG SUVRs in control mice. We note that the TD mouse model we used is an alimentary TD model rather than the intraperitoneal pyrithiamine (PT) model (with alimentary thiamine deficiency [PT model]) typically used [30]. Under the TD model, mice can live for over 28 days, whereas in the PT model, mice typically live for less than 14 days. Furthermore, the TD model exhibits brain damage in multiple brain regions similar to AD, whereas the PT model manifests typical thalamus lesions, similar to human Wernicke’s encephalopathy pathology [30]. We used the TD model in our study because we believe that it more closely mimics the condition in human patients with AD. Both brain and blood TDP levels were tightly correlated with FDG SUVRs in multiple brain regions in mice (Fig. 2). Previous studies have demonstrated that TDP reduction is a significant and common feature in patients with AD [4, 21]. Thus, we further investigated the correlation between blood TDP levels and brain glucose hypometabolism in patients with AD. The results verified the findings in mice, with blood TDP levels positively correlating with FDG SUVRs in multiple observed brain regions of patients with AD and in the combined group (Fig. 1). To our knowledge, our study is the first to disclose that TDP reduction is a potential mediator of brain glucose hypometabolism in AD.

Brain Aβ deposition is a prominent pathological characteristic and is considered as an initial factor in the AD pathophysiological cascade. However, previous studies have demonstrated no obvious direct correlation between brain Aβ accumulation and glucose hypometabolism in AD [7–11], and our study also supports the lack of such correlations (Fig. 5).

In order to exclude the indirect effect of brain Aβ deposition on brain glucose metabolism by disturbing thiamine metabolism, we analyzed the relationship between brain Aβ deposition and thiamine metabolism. We found that blood TDP levels in patients with AD did not significantly correlate with brain Aβ deposition as assayed by PiB-PET (Fig. 3). This result was further tested in APP/PS1 transgenic mice, which did not exhibit significant changes in the levels of brain and blood thiamine metabolites, although Aβ deposition was significant in brains of 6- to 12-month-old APP/PS1 transgenic mice (Fig. 6). We also ruled out the possibility of an indirect effect of Aβ deposition on enhancing the vulnerability of brain glucose metabolism to TD. TD significantly impaired brain FDG uptake in both APP/PS1 transgenic mice and age-matched wild-type mice. However, there were no significant differences in the changes in glucose uptake in representative brain regions between APP/PS1 transgenic and wild-type mice following TD (Fig. 4). These results show that Aβ deposition did not enhance the susceptibility of brain glucose metabolism to TD.

Glucose is a dominant substrate of brain energy metabolism, on which brain function absolutely depends. Currently, brain glucose hypometabolism in AD is generally considered a consequence of synaptic and neuronal dysfunction or loss; in other words, because there are fewer neurons, the brain has lower energy demand. However, the brain, as the organ with the highest energy consumption in the body, is vulnerable to dyshomeostasis of glucose metabolism. Thus, an alternative hypothesis is that the disturbance of glucose metabolism and its pathogenic factor(s) leads to synaptic and neuronal dysfunction and neurodegeneration in AD [4].

TDP, the active form of thiamine, is a critical coenzyme of three key enzymes in glucose metabolism: pyruvate dehydrogenase and α-ketoglutarate dehydrogenase in the Krebs cycle and transketolase in the pentose phosphate pathway. Glucose metabolism takes place predominantly in mitochondria, and mitochondrial dysfunction has been well demonstrated as an early event in AD [31, 32]. Thus, it is possible that mitochondrial dysfunction contributes to brain glucose hypometabolism. The identification of causal factor(s) of mitochondrial dysfunction could help identify the real culprit of brain glucose hypometabolism. As a critical coenzyme of glucose metabolism-related mitochondrial enzymes (pyruvate dehydrogenase and α-ketoglutarate dehydrogenase), TDP obviously has an important effect on mitochondria. Thus, we can reasonably speculate that TDP reduction induces mitochondrial dysfunction in AD.

A disease-modifying drug for AD is still lacking. Given that amyloid plaques were also observed in elderly persons who did not show pathological cognitive impairments and in many clinical trials targeting the reduction of brain Aβ deposition, and given that inhibition of tau aggregation was found to have little effect on AD cognitive decline and progression [33–36], new therapeutic strategies for AD are urgently needed. Our study indicates that a new therapeutic strategy that simultaneously targets abnormal thiamine metabolism and multiple disease-causing mechanisms should be explored. Our preliminary results derived from an open, noncontrol clinical observation with a small sample show long-term beneficial effects of benfotiamine, a thiamine derivate with multifaceted pharmacological effects [37, 38], on cognitive deterioration of patients with AD with positive PiB-PET imaging. The beneficial effect is independent of brain Aβ deposition evaluated by PiB-PET imaging [39].

There are still many unanswered questions to be clarified in brain glucose hypometabolism of AD. First, the dysfunction of glucose metabolism in brains of patients with AD is regionally selective and exhibits an orderly temporal and spatial pattern. What is the mechanism, and is it linked to different vulnerabilities of types of neurons to metabolic demands? Second, although clinical studies have indicated that brain glucose hypometabolism precedes by decades clinical symptoms and brain atrophy in both sporadic and familial AD [12, 13, 40], implying no significant correlation with the loss of neurons and synapses, we still lack direct evidence to decode the relationship between brain glucose hypometabolism due to TDP reduction and AD-type neurodegeneration. Which is the cause? Which is the consequence? Third, does brain glucose hypometabolism of patients with sporadic and familial AD go through different mechanisms or share a common pathogenesis? Fourth, is brain glucose hypometabolism due to TDP reduction associated with other pathophysiological features of AD, such as tau hyperphosphorylation, neuroinflammation, insulin resistance, and microvascular dysfunction? Finally, our previous study showed that enhanced activities of phosphatases contribute to TDP reduction in AD [41]. What is the mechanism? The answers to these questions will promote understanding of AD pathogenesis and help in the exploration of new approaches to modifying the disease.

## Conclusions

For the first time, we demonstrate in vivo that TDP reduction strongly correlates with brain glucose hypometabolism, whereas amyloid deposition does not. Our study demonstrates a novel mechanism that can lead to brain glucose hypometabolism in AD, a mechanism that may be a useful target for disease-modifying therapy.

## Additional file

Additional file 1: **Figure S1.** Blood TDP levels do not significantly correlate with brain glucose metabolism in control subjects. **Figure S2.** Blood TMP and thiamine levels do not significantly correlate with brain amyloid deposits in patients with AD. **Figure S3.** Blood and brain TDP levels do not significantly correlate with brain glucose metabolism in control mice. **Figure S4.** Blood TMP and thiamine levels do not significantly correlate with brain amyloid deposits in patients with AD. (PDF 454 kb)

## Abbreviations

Aβ: β-Amyloid
AD: Alzheimer’s disease
ADL: Activities of daily living
ANOVA: Analysis of variance
APOE: Apolipoprotein E
APP/PS1: Amyloid precursor protein/presenilin-1
CDR: Clinical Dementia Rating
CT: Computed tomographic
CTX: Cortex
ELISA: Enzyme-linked immunosorbent assay
FDG-PET: Positron emission tomography with 2-[^18^F]fluoro-2-deoxy-d-glucose
MMSE: Mini Mental State Examination
PET: Positron emission tomography
PiB: ^11^C-Pittsburgh compound B
PT: Pyrithiamine
RHIP: Right hippocampus
ROI: Region of interest
RSTR: Right striatum
SUVR: 2-[^18^F]fluoro-2-deoxy-d-glucose standardized uptake value ratio
TD: Thiamine deficiency
TDP: Thiamine diphosphate
THA: Thalamus
TMP: Thiamine monophosphate
WT: Wild type
