# Supplementary material for: Thiamine diphosphate reduction strongly correlates with brain glucose hypometabolism in Alzheimer’s disease, whereas amyloid deposition does not
Source: Alzheimers Res Ther. 2018 Mar 1;10:26. doi: 10.1186/s13195-018-0354-2 (PMC5831864; doi:10.1186/s13195-018-0354-2)
Supplement: Supplementary file 1 — Figure S1. Blood TDP levels do not significantly correlate with brain glucose metabolism in control subjects. Figure S2. Blood TMP and thiamine levels do not significantly correlate with brain amyloid deposits in patients with AD. Figure S3. Blood and brain TDP levels do not significantly correlate with brain glucose metabolism in control mice. Figure S4. Blood TMP and thiamine levels do not significantly correlate with brain amyloid deposits in patients with AD. (PDF 454 kb) [file 13195_2018_354_MOESM1_ESM.pdf]

Thiamine diphosphate reduction strongly correlates with brain glucose hypometabolism in Alzheimer's disease, while amyloid deposition does not

Shaoming Sang[1#], Xiaoli Pan[1#], Zhichun Chen[1], Fan Zeng[5], Shumei Pan[1], Huimin Liu[1], Lirong Jin[1], Guoqiang Fei[1], Changpeng Wang[1], Shuhua Ren[3], Fangyang Jiao[3], Weiqi Bao[3], Weiyan Zhou[3], Yihui Guan[3], Yiqiu Zhang[4], Hongcheng Shi[4], Yanjiang Wang[5], Xiang Yu[2], Yun Wang[1]*, Chunjiu Zhong[1]*

Supplementary Figures

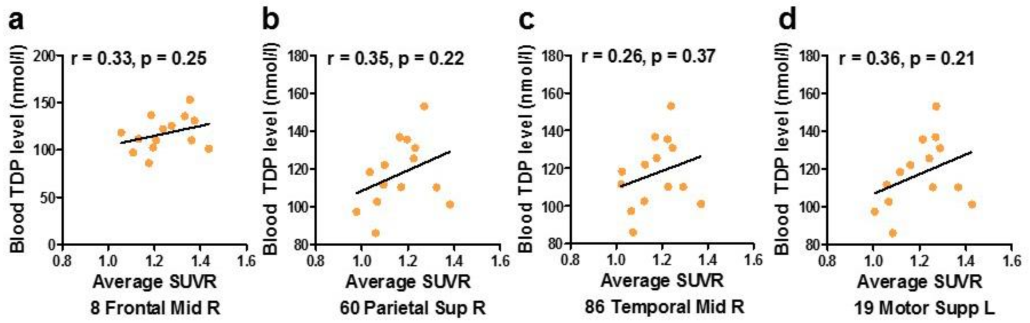

Figure S1. Blood TDP levels do not significantly correlate with brain glucose metabolism in control subjects. a-d. Correlation between SUVRs of representative brain regions and blood TDP levels in control subjects (N = 14).

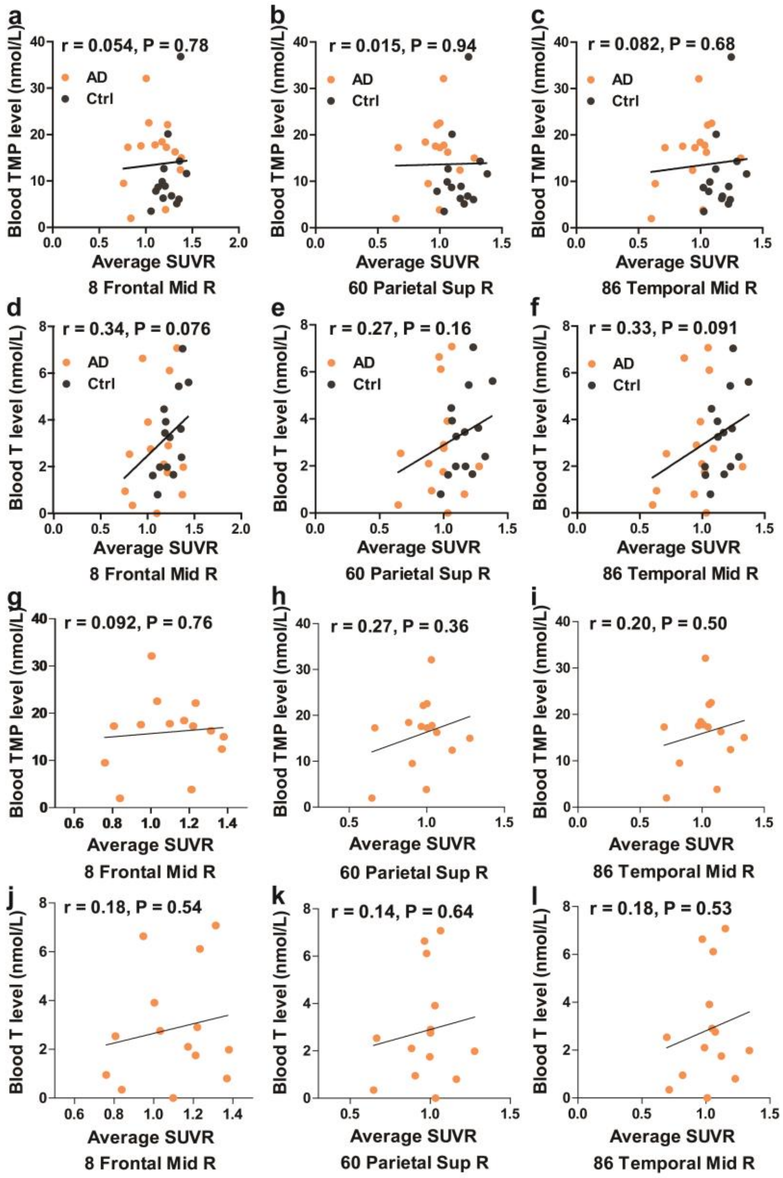

Figure S2. Blood TMP and thiamine levels do not significantly correlate with brain glucose metabolism. a-c. Correlation between SUVRs of representative brain regions and blood TMP levels in all subjects (N = 28). **d-f.** Correlation between SUVRs of representative brain regions and blood thiamine levels in all subjects (N = 28). **g-i.** Correlation between SUVRs of representative brain regions and blood TMP levels in AD patients (N = 14). **j-l.** Correlation between SUVRs of representative brain regions and blood thiamine levels in AD patients (N = 14).

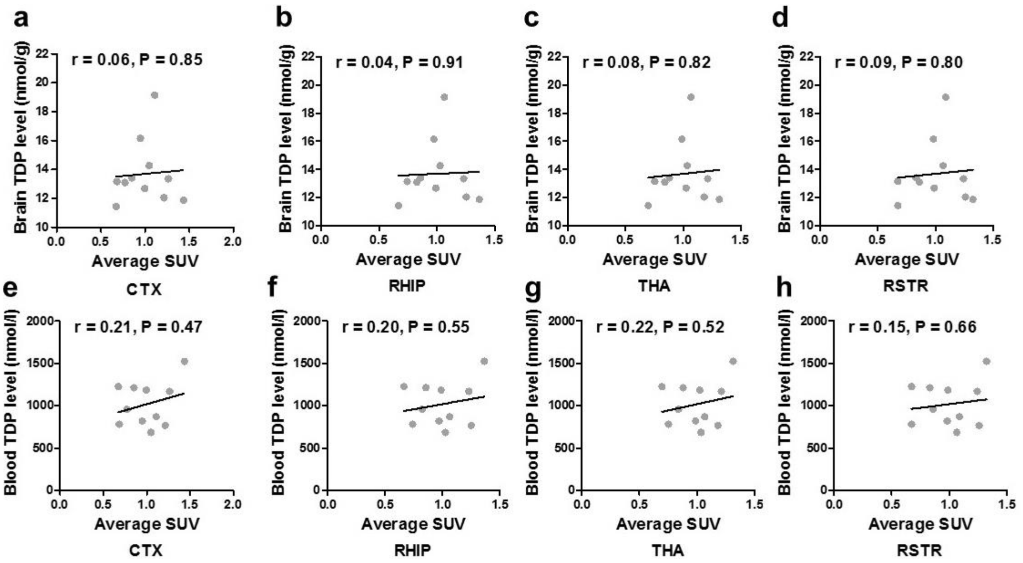

Figure S3. Blood and brain TDP levels do not significantly correlate with brain glucose metabolism in control mice. a-d. Correlations between brain TDP levels and SUVs in representative brain regions of control mice (N = 11). **e-h**. Correlations between blood TDP levels and SUVs in representative brain regions of control mice (N = 11).

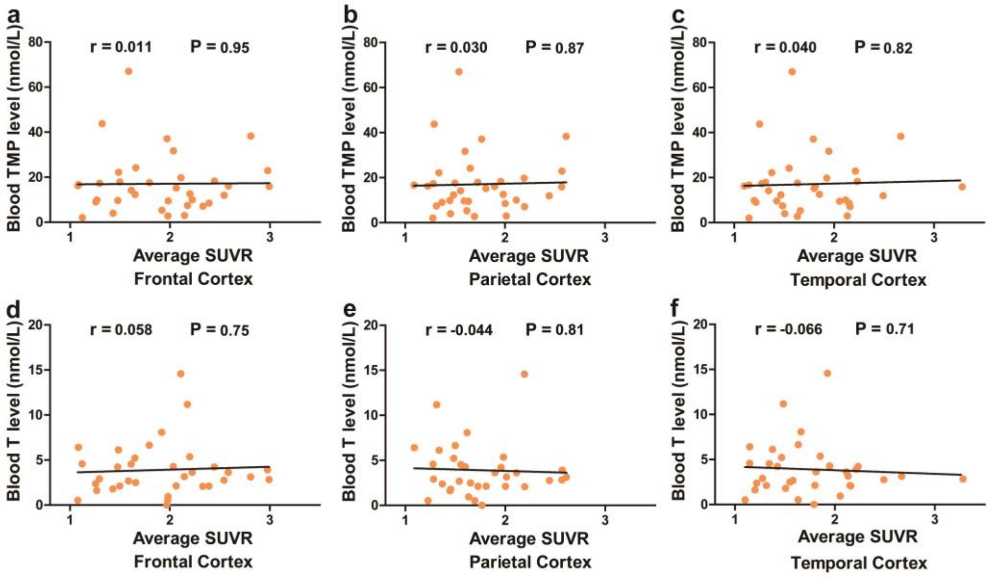

Figure S4. Blood TMP and thiamine levels do not significantly correlate with brain amyloid deposits in AD patients. a-c. Blood TMP levels did not significantly correlate with average SUVRs of representative brain regions in AD patients (N = 35). **d-f**. Blood thiamine levels did not significantly correlate with average SUVRs of representative brain regions in AD patients (N = 35).
